# Beneficial effects of a cognitive-behavioral occupational stress management group training: the mediating role of changing cognitions

**DOI:** 10.3389/fpsyg.2023.1232172

**Published:** 2024-07-29

**Authors:** Petra H. Wirtz, Alisa Auer, Norbert K. Semmer, Ulrike Ehlert, Fridtjof W. Nussbeck

**Affiliations:** ^1^Biological Work and Health Psychology, University of Konstanz, Konstanz, Germany; ^2^Centre for the Advanced Study of Collective Behaviour, University of Konstanz, Konstanz, Germany; ^3^Psychology of Work and Organizations, Department of Psychology, University of Bern, Bern, Switzerland; ^4^Department of Clinical Psychology and Psychotherapy, University of Zurich, Zurich, Switzerland; ^5^Methods for Intensive Data in Psychology, University of Konstanz, Konstanz, Germany

**Keywords:** cognitive-behavioral stress management training, cognitive restructuring, perceived mastery of functional stress management skills, exhaustion, job dissatisfaction, trait anger, group training

## Abstract

**Introduction:**

While the effectiveness of cognitive-behavioral stress management trainings (SMTs) is well-documented, the underlying mechanisms, especially in an occupational context, are not fully understood. We tested whether SMT-induced improvements in stress management skills, particularly in the mastery of changing cognitions, may explain beneficial SMT effects.

**Methods:**

Our non-randomized controlled trial comprised 108 employees of a German health insurance company, with 65 of them participating in a cognitive-behavioral SMT and 43 participating in an alternative control training (AT). As outcome variables, we repeatedly assessed stress-related (functional stress management skills, relaxation, stress reactivity, exhaustion), work-related (job dissatisfaction), and specific-context-related (social support, trait anger) measures at baseline, 2 weeks, and 3 months after the trainings. Functional stress management skills and, in particular, a subscale assessing perceived mastery of changing cognitions (“cognitive-strategies-and-problem-solving”) were tested as mediators of change.

**Results:**

Repeated measures (M)AN(C)OVAs and complementary multigroup latent difference models confirmed improvements in all outcomes in the SMT-group compared to the AT-group (*p'*s ≤ 0.015). Multivariate mediation path analyses revealed that, regarding mechanisms of change, the subscale cognitive-strategies-and-problem-solving was identified as the most important mediator for all outcomes (95% CIs for expected increases in SMT- vs. AT-group = [lower limits (LLs) ≥ 0.004]; 95% CIs for expected decreases in the SMT- vs. AT-group = [upper limits(ULs) ≤ −0.078]) except for job dissatisfaction.

**Discussion:**

Our findings confirm that employees can effectively learn to master stress reduction techniques and consequently lower the resulting burden. Moreover, beneficial SMT effects seem to result from improvements in functional stress management skills, particularly in the ability to change cognitions. This points to the importance of training cognitive techniques.

## 1 Introduction

Stress is a major risk factor for health impairments that causes substantial financial burden and affects individuals, organizations, and societies (e.g., Brotman et al., 2007; Hassard et al., 2014, 2017). Adverse health consequences of chronic stress exposure, in particular, in the context of work, include not only exhaustion but also increased risk, both for somatic diseases, such as cardiovascular disease, and for mental diseases (Appels, 1999; Stansfeld and Candy, 2006; Aronsson et al., 2017; Kivimäki and Steptoe, 2018).

**Figure 1:**
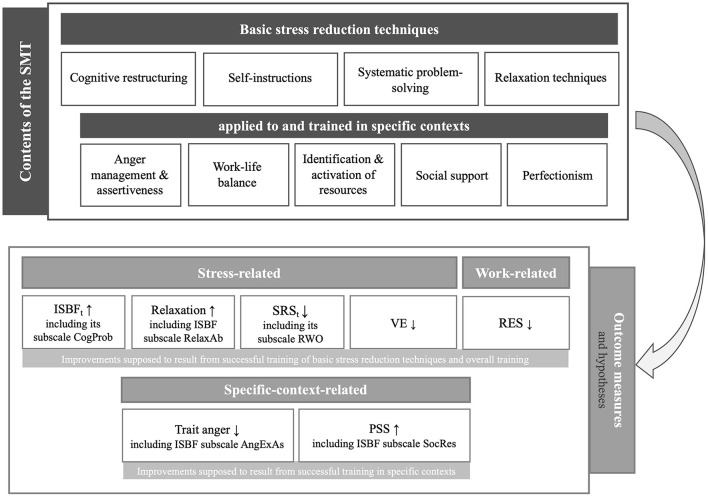
Content and structure of the stress management training (SMT) related to hypothesized changes in stress-related, work-related, and specific-context-related outcome measures. ISBF_t_, Inventory for Assessment of Stress Management Skills total-score; Relaxation, Relaxation after work; SRS_t_, Stress-Reactivity-Scale total-score; VE, vital exhaustion; RES, resigned attitude toward one's job; PSS, perceived social support; CogProb, ISBF subscale cognitive-strategies-and-problem-solving; RelaxAb, ISBF subscale relaxation-abilities; RWO, SRS subscale reactivity-to-work-overload; AngExAs, ISBF subscale adequate-anger-expression-and-assertiveness; SocRes, ISBF subscale identification-and-use-of-social-resources; ↑, hypothesized increases; ↓, hypothesized decreases.

Stress management interventions aim to reduce stress and the resulting burden. Evidence from meta-analyses confirms the high effectiveness of person-focused stress management trainings (SMTs) with cognitive-behavioral elements (Saunders et al., 1996; Van der Klink et al., 2001; Richardson and Rothstein, 2008; Kröll et al., 2017). The core element of cognitive-behavioral SMTs are cognitive techniques, including cognitive restructuring (Ellis, 1962; Beck, 1967) and resulting self-instructions (Meichenbaum, 1985, 2017), but also systematic problem solving (e.g., D'Zurilla and Nezu, 1982). Cognitive techniques are often combined with relaxation techniques such as progressive muscle relaxation (PMR) (Jacobsen, 1929), as in the well-established Stress Inoculation Training (SIT) by Meichenbaum (1985). Moreover, in later cognitive-behavioral SMT manuals, these techniques were trained in specific contexts such as anger, work–life balance, or social context (e.g., Siegrist and Silberhorn, 1998; Wiegard et al., 2000; Williams and Williams, 2010; Kaluza, 2018a,b). Other SMTs use cognitive techniques more or less explicitly when working on changes in perceptions and cognitions that consequently result in increased relaxation or calmness (Kabat-Zinn, 1982, 1990, 1994; Bond and Hayes, 2002; Bond, 2004). Our occupational cognitive-behavioral SMT included the basic stress reduction techniques of cognitive restructuring, self-instructions, systematic problem-solving, and relaxation techniques based on the Stress Inoculation Training by Meichenbaum (1985). In line with other occupational stress reduction programs, these techniques were applied to and trained in stress-relevant specific contexts, including anger management and assertiveness (Siegrist and Silberhorn, 1998; Wiegard et al., 2000), work–life balance (Kaluza, 1996; Reschke and Schröder, 2000; Wagner-Link, 2001), identification and activation of resources (Siegrist and Silberhorn, 1998), social support (Reschke and Schröder, 2000; Wagner-Link, 2001), and perfectionism (Siegrist and Silberhorn, 1998; Wiegard et al., 2000) (see Figure 1).

The effectiveness of SMTs has been examined regarding a broad range of psychological (e.g., stress, coping, general mental health, work satisfaction, social support), physiological (e.g., blood pressure, stress hormones), and organizational outcome measures (e.g., absenteeism, job performance) (c.f., Van der Klink et al., 2001; Richardson and Rothstein, 2008; Kröll et al., 2017; Riley et al., 2017). Overall, meta-analyses revealed medium to large effect sizes for beneficial effects of cognitive-behavioral SMTs, in particular, for psychological measures (Saunders et al., 1996; Van der Klink et al., 2001; Richardson and Rothstein, 2008; Kröll et al., 2017). In our study, we assessed psychological measures that we expected to be improved by our SMT based on its contents (i.e., basic stress reduction techniques and their training in stress-relevant specific contexts; see Figure 1). More precisely, the assessed psychological outcome measures comprise *stress-related measures*, i.e., perceived mastery of functional stress management skills, including subscales, relaxation after work, stress reactivity (in particular, to work overload), vital exhaustion (VE), and the *work-related measure* job dissatisfaction. In addition, we included *specific-context-related measures*, i.e., perceived social support (PSS) and trait anger. Notably, we expected improvements in stress- and work-related measures to result from successful training of the basic stress reduction techniques and the overall training, while we expected the specific-context-related measures to improve, in particular, from successful training of the basic stress reduction techniques in the respective specific contexts.

While the effectiveness of cognitive-behavioral SMTs is well-documented, the underlying psychological mechanisms are not fully understood. The transactional model of stress by Lazarus and Folkman (1984) can help to understand the psychological mechanisms underlying effective, successful cognitive-behavioral SMTs. According to the transactional model of stress, a stress reaction results from the perceived imbalance between situational demands and personal resources based on cognitive appraisal processes, with primary appraisal addressing harm, threat, and challenge of a situation and secondary appraisal addressing the person's coping options (Lazarus and Folkman, 1984). Accordingly, changing the cognitive appraisal of a stressful situation by means of cognitive techniques should change the resulting stress experience. Indeed, based on quantitative assessment, such as the frequency of using a certain coping strategy, there is emerging evidence that coping in terms of *changing cognitions* may play a role in mediating the beneficial SMT effects on stress and mental health outcomes (Bond and Bunce, 2000; Gaab et al., 2003; Hammerfald et al., 2006; Keogh et al., 2006; Flaxman and Bond, 2010; Leung et al., 2010; Brinkborg et al., 2011; Lloyd et al., 2013). However, according to the framework of moderators, mediators, and mechanisms of change of occupational SMTs proposed by Bunce (1997), changes in terms of *mastery* and, thus, quality of coping are relevant to induce beneficial SMT outcomes.

A standardized questionnaire for the assessment of *perceived mastery of functional stress management skills* (Wirtz et al., 2013) was validated previously based on the Measure-of-Current-Status (MOCS) by Carver (2006), both including a subscale that assesses the perceived mastery of changing cognitions using cognitive strategies such as problem-solving. To date, the mediation effects of perceived mastery of changing cognitions on beneficial psychological outcomes have only been investigated in one study, notably in the context of cancer and in an individual therapeutic setting. In that study, Marsland et al. (2020) reported mediating effects via perceived mastery of changing cognitions in addition to mediating effects via MOCS-total-score on intervention-induced improvements in perceived stress and anxiety in mothers of children who were newly diagnosed with cancer. Notably, the cognitive-behavioral therapeutic intervention was specifically tailored for cancer caregivers with a special focus on coping with cancer. This finding is in line with a study in cancer patients that found SMT-induced improvements in quality of life and in benefit-finding from cancer to be mediated by changes in the MOCS-total-score (Penedo et al., 2004, 2006). So far, several questions regarding the generalization of these findings remain unclear. First, it needs to be elucidated whether mediating effects of functional stress management skills and changing cognitions can be generalized to a broader range of outcome measures, including further stress-related outcome measures, but also work-related and more specific outcome measures, such as anger and social support. Second, it remains unclear whether the findings of a specifically tailored therapeutic intervention can be extended to occupational SMTs, i.e., SMTs targeting stress management on an occupational and non-cancer-specific basis. This would be of particular importance in the context of health prevention, given the above-outlined adverse health effects of occupational stress. Third, it is unclear whether findings, in particular, with regard to changing cognition of the individual therapeutic setting, can be extended to a group-based SMT. Given that group-based trainings would allow a substantial increase in efficiency by helping more people within the same time with the given resources, it would be of particular importance to show that group-based trainings are capable of changing cognitions that in turn mediate effectiveness of the trainings. Based on this reasoning, we aimed at investigating perceived mastery of functional stress management skills and its subscales not only as a stress-related outcome variable but also by considering significant intervention-induced improvements in the respective scales as mediators of beneficial SMT effects.

Taken together, in the current study, we investigated the effects and underlying psychological mechanisms of an occupational cognitive-behavioral SMT compared to alternative control trainings (ATs) over a period of 3 months. Figure 1 provides a structured overview of contents and outcome measures of our SMT including specific hypotheses regarding SMT effects on outcome measures. We hypothesized that the SMT, on the one hand, would increase the stress-related measures of perceived mastery of functional stress management skills and relaxation after work as well as the specific-context-related measure of PSS compared to AT. On the other hand, we hypothesized that the SMT would reduce the stress-related measures of VE and stress reactivity (in particular, to work overload), the work-related measure of job dissatisfaction, and the specific-context-related measure of trait anger compared to AT (see Figure 1). Moreover, with respect to underlying mechanisms, we expected that intervention-induced increases in perceived mastery of functional stress management skills in general and in the subscales assessing specific stress management skills would mediate the hypothesized positive effects of the SMT. Given the importance of cognitions in the context of stress (Lazarus and Folkman, 1984), we specifically hypothesized that increases in the stress management skill subscale “perceived master of changing cognitions” would mediate improvements in all outcome variables.

## 2 Materials and methods

### 2.1 Study participants and procedure

We conducted a field study in employees (aged 18–65 years) of a German health insurance company located all over Germany. Parts of this study were used for psychometric validation of the Inventory for Assessment of Stress Management Skills (Wirtz et al., 2013) and for a doctoral thesis (Stein, 2007) (for more information, see Supplementary material 1). The study was carried out in accordance with the Declaration of Helsinki principles and was formally approved by the company's board of management and staff council. All participants provided written informed consent.

**Figure 2:**
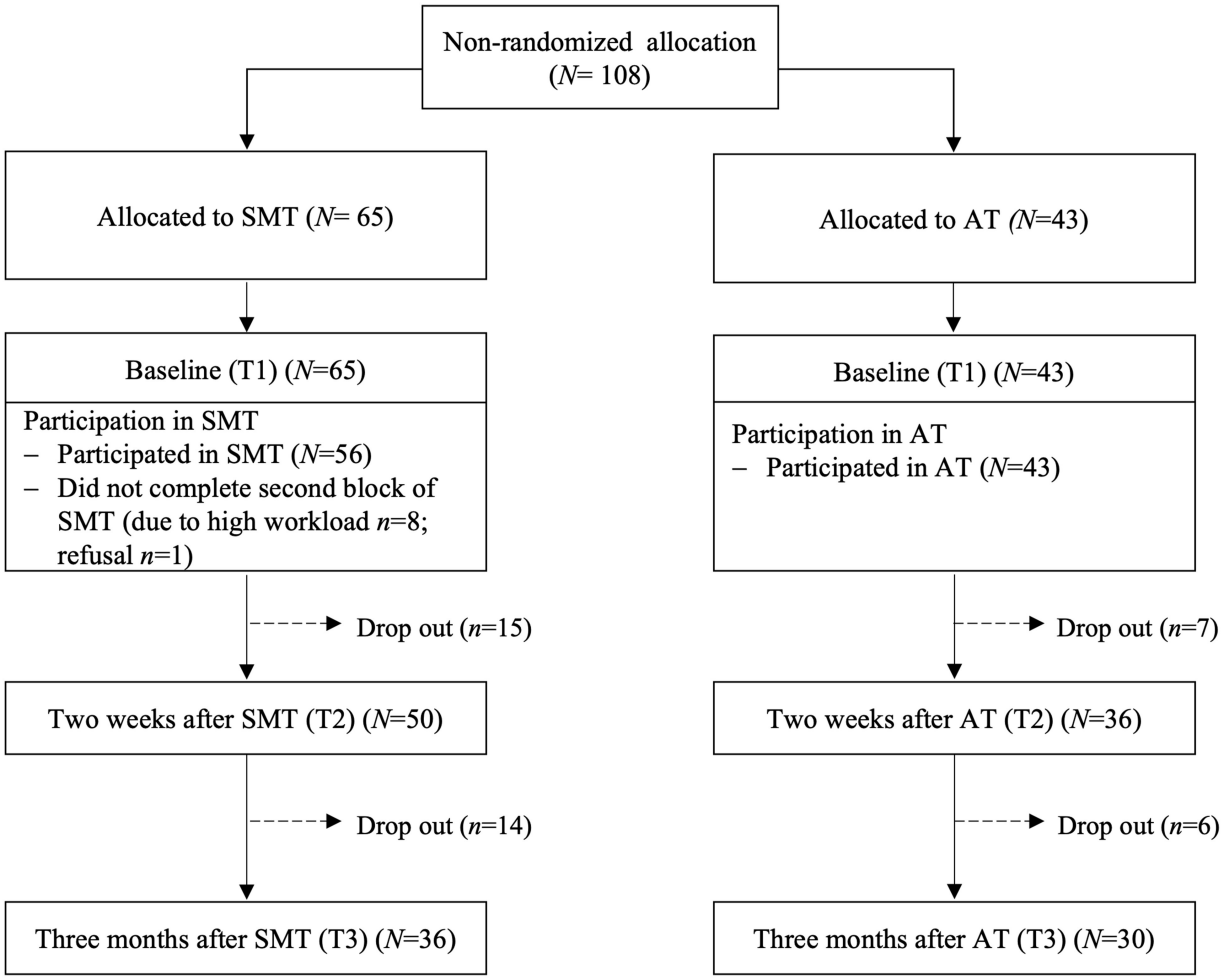
Participants' allocation to study groups and participant flow through the study. SMT, stress management training; AT, alternative training; *N*, sample size; T, timepoint.

A non-randomized controlled trial with an active control group was conducted in Germany. As personnel development procedures on a voluntary basis, the company offered its employees participation in different group trainings, including our cognitive-behavioral SMT. Our control group underwent one of two ATs, either on the structure and organization of the company and its corporate guidelines (“Basic Training”) or on working strategies with respect to communication and cooperation (“Working efficiently”). None of the ATs were explicitly related to stress or stress management. Outcome measures were assessed at three points in time (T) in all training groups: at baseline, i.e., immediately before the beginning of the respective training (“baseline,” i.e., T1), at 2 weeks (“post,” i.e., T2), and at 3 months (“follow-up,” i.e., T3) after the end of the training. For the experimental *SMT-group*, attendees of seven SMT groups were asked to participate in the study. All 65 attendees volunteered for the study and participated in the baseline assessment (*N* = 65). A total of 56 participants finished both parts of the SMT. Reasons for not attending the second part of the SMT and thus completing the training were being affected by a disease or a high workload (*n* = 8) and one participant denied to participate. Post assessment data were provided by 50 participants and follow-up assessment data were provided by 36 participants. For the *AT-group*, participants of the personnel development trainings “Basic training” and “Working efficiently” taking place during a time-frame close to the SMTs were asked for participation. A total of 43 participants volunteered to participate in the AT-group at baseline, with 36 persons providing post assessment and 30 persons completing follow-up assessment. Participants' allocation to the study groups and participants' flow through the study are depicted in Figure 2.

### 2.2 Intervention

Our occupational SMT addresses basic stress reduction techniques and their application and training in specific (mostly work-related) contexts using cognitive-behavioral techniques. We specifically tailored a group-oriented 4-day training that consisted of two blocks. Each block was conducted over 2 consecutive workdays with a 4-week time interval between the first and the second block to allow for individual training and “homework.” Group sizes varied between 7 and 12 participants, with two trainers per group. As already outlined in the introduction, the SMT addressed the basic stress reduction techniques of cognitive restructuring, self-instructions, systematic problem-solving, and relaxation techniques, based on the Stress Inoculation Training by Meichenbaum (1985). In line with other occupational stress reduction programs, these techniques were applied to and trained in stress-relevant specific contexts including anger management and assertiveness (Siegrist and Silberhorn, 1998; Wiegard et al., 2000), work–life balance (Kaluza, 1996; Reschke and Schröder, 2000; Wagner-Link, 2001), identification and activation of resources (Siegrist and Silberhorn, 1998), social support (Reschke and Schröder, 2000; Wagner-Link, 2001), and perfectionism (Siegrist and Silberhorn, 1998; Wiegard et al., 2000) (see Figure 1). Each technique or topic, respectively, was introduced by brief theoretical inputs to allow for a maximum of supervised stress management practice during the training based on participants' stress problems.

### 2.3 Measures

Given the potential confounding effects of baseline work stress, anxiety, and depression on presumed changes in outcome variables over time, we assessed these variables at baseline (T1) to test for group differences in addition to sociodemographic data. As outcome variables, we repeatedly assessed the stress-related measures of perceived mastery of functional stress management skills (total-score and subscales), relaxation after work, stress reactivity (total-score and subscale reactivity-to-work-overload), and VE, the work-related measure of job dissatisfaction, and the specific-context-related measures of PSS and trait anger at baseline (T1), as well as 2 weeks (T2) and 3 months (T3) after the end of the training. Notably, perceived mastery of functional stress management skills (total-score and subscales) were first tested as outcome measures when analyzing changes over time in the study groups. Second, significant training-induced improvements in these scales were further tested as mediators of change in the mediation models.

#### 2.3.1 Baseline-only assessment: work stress, anxiety, and depression

Effort-reward imbalance (ERI) assesses stressful experiences at work with a questionnaire consisting of two scales measuring perceived effort (scale effort: 5 items) and experienced or anticipated rewards (scale reward: 11 items) (Siegrist, 1996; Rödel et al., 2004; Siegrist et al., 2004). The ratio of effort and reward was calculated according to established recommendations (Siegrist et al., 2004), yielding a quantitative estimate of the effort–reward imbalance, with higher values indicating a higher degree of stressful experience at work. Cronbach's α (*N* = 102) in our sample was 0.74 for “effort” and 0.80 for “reward.” Anxiety was assessed by the 20-item trait version of the state-trait-anxiety-inventory (STAI) (Laux et al., 1981), with higher scores indicating higher anxiety. The extent of depression was assessed with 15 items by the short version of the “Allgemeine Depressionsskala” (ADS-K) (Hautzinger and Bailer, 1993), with higher values indicating higher depressive symptomatology. The ADS-K is the German version of the “Center for Epidemiological Studies Depression Scale” (CES-D) (Radloff, 1977). In our sample, Cronbach's α was 0.87 for anxiety (*N* = 93) and 0.86 for depression (*N* = 94).

#### 2.3.2 Stress-related measures

##### 2.3.2.1 Mastery of functional stress management skills

We used the 14-item Inventory for Assessment of Stress Management Skills (German “Inventar zur Erfassung von Stressbewältigungsfertigkeiten,” ISBF) to assess perceived mastery of functional stress management skills (Wirtz et al., 2013). We computed the total-score and the following five subscales “cognitive-strategies-and-problem-solving” (corresponding to the MOCS-scale “coping confidence”; Carver, 2006), “adequate-anger-expression-and-assertiveness,” “identification-and-use-of-social-resources,” “relaxation-abilities,” and “perception-of-bodily-tension.” Notably, the items of the subscale “cognitive-strategies-and-problem-solving” assess perceived mastery of changing cognitions using cognitive strategies, including strategies to solve occurring problems. Participants are asked to rate on a 5-point response scale how well they can perform on each of the items (e.g., “I can easily stop and re-examine my thoughts to gain a new perspective” (German: “Ich kann meine Gedanken leicht stoppen und überprüfen, um zu neuen Perspektiven zu gelangen”); 1 = I cannot do this at all to 5 = I can do this extremely well). Higher scores indicate better mastery of functional stress management skills. Reliability (Cronbach's α (*N* = 332) = 0.83) and validity were found to be adequate (Wirtz et al., 2013) with comparable Cronbach's α (*N* = 108) = 0.82 in our sample at baseline.

##### 2.3.2.2 Relaxation after work

Relaxation after work was assessed by the 4-item subscale of the Recovery Experience Questionnaire that measures unwinding and recuperation from work during leisure time (Sonnentag and Fritz, 2007). Relaxation, as assessed by this questionnaire, refers to a process often associated with leisure activities characterized by a state of low activation and increased positive affect. Participants rated all items on a 5-point response scale (e.g., “I do relaxing things” (German: “Am Feierabend unternehme ich Dinge, bei denen ich entspannen kann”); 1 = I do not agree to 5 = I fully agree). Items are summed up to a total-score, with higher scores indicating higher relaxation after work. Psychometric properties were found to be adequate with Cronbach's α (*N* = 930) = 0.85 for the relaxation subscale (Sonnentag and Fritz, 2007) and comparable with Cronbach's α (*N* = 108) = 0.88 in our sample at baseline.

##### 2.3.2.3 Stress reactivity

Subjective stress reactivity was measured using the Stress-Reactivity-Scale (SRS) (Schulz et al., 2005). The SRS comprises 29 items forming six scales that assess the intensity of different aspects of stress reactions to different types of stress situations. The scales are summarized to a total-score assessing general stress reactivity, with higher scores indicating higher stress reactivity. Each item describes a potentially stressful situation (e.g., “When I have little time for a job to be done…” (German: “Wenn ich für meine Arbeit wenig Zeit habe…”)) and asks participants to choose their typical response out of three potential responses depending on the potentially stressful situation (e.g., “I usually stay calm”, “I usually feel uneasy”, “I usually get quite agitated” (German: “bleibe ich meinst ruhig”, “werde ich meist unruhig”, “werde ich meist ziemlich hektisch”)). In this study, we used the SRS-total-score and the subscale “reactivity-to-work-overload.” Reliability (Cronbach's α (*N* = 975) = 0.91) and validity were found to be adequate (Schulz et al., 2005) with comparable Cronbach's α (*N* = 108) = 0.90 in our sample at baseline.

##### 2.3.2.4 Vital exhaustion

We assessed VE using the German version of the short form of the Maastricht Exhaustion Questionnaire (Kopp et al., 1998; Wirtz et al., 2003; Kudielka et al., 2004). Nine items that are summarized to a total-score ask about the presence or absence of undue tiredness, trouble falling asleep, repeated waking up at night, general malaise, listlessness, irritability, loss of energy, demoralization, and waking up exhausted (e.g., “Do you often feel tired?” (German: “Fühlen Sie sich oft müde?”); 2 = yes, 1 = I don't know, 0 = no). Higher scores indicate higher VE. Psychometric properties of the short form of the Maastricht Exhaustion Questionnaire have been shown to be adequate with Cronbach's α (*N* = 822) = 0.84 (Kudielka et al., 2004). In our sample, Cronbach's α (*N* = 105) was 0.84 at baseline.

#### 2.3.3 Work-related measures

##### 2.3.3.1 Job dissatisfaction

As a measure of job dissatisfaction, we measured a resigned attitude toward one's job, i.e., an attitude of resigned and resentful acceptance of unpleasant conditions at work, with four items (Baillod and Semmer, 1994; Grebner et al., 2005). Participants were asked to rate on a 7-point response scale how often they have thoughts such as “My job is not ideal, but it could be worse.” (German: “Meine Arbeit ist zwar nicht gerade ideal, aber schließlich könnte sie noch schlimmer sein”; 1 = never to 7 = always). Items are summarized to a total-score with higher scores indicating a higher resigned attitude toward one's job. The internal consistency was found to be between Cronbach's α = 0.68 (Baillod and Semmer, 1994) and Cronbach's α = 0.72 (Grebner et al., 2005) with Cronbach's α (*N* = 108) = 0.65 in our sample at baseline.

#### 2.3.4 Specific-context-related measures

##### 2.3.4.1 Perceived social support

PSS was assessed by the 8-item subscale of the Berlin Social Support Scale (BSSS) (Schulz and Schwarzer, 2003). Participants were asked to rate their agreement with statements such as “There are people that offer me help when I need it” (German: “Es gibt Menschen, die mir Hilfe anbieten, wenn ich sie brauche”) on a 4-point response scale (1 = disagree to 4 = agree). Items were averaged to compute the PSS score, with higher scores indicating higher PSS. Psychometric properties were found to be adequate with Cronbach's α (*N* = 437) = 0.83 for the PSS subscale (Schulz and Schwarzer, 2003) and comparable Cronbach's α (*N* = 108) = 0.91 in our sample at baseline.

##### 2.3.4.2 Trait anger

We used the 10-item trait anger subscale of the German version of the State Trait Anger Expression Inventory (STAXI) to assess participant's disposition to experience anger (Schwenkmezger et al., 1992). Participants were asked to rate themselves with items such as “I am quick tempered” (German: “Ich werde schnell ärgerlich”) on a 4-point response scale (1 = never to 4 = almost always). Items were summarized to a total-trait-anger-score with higher scores, indicating a greater level of trait anger. Psychometric properties were found to be adequate with Cronbach's α (*N* = 990) = 0.71 (Schwenkmezger et al., 1992) and good with Cronbach's α (*N* = 107) = 0.84 in our sample at baseline.

### 2.4 Statistical analyses

We conducted our analyses with SPSS (Version 28.0; IBM SPSS Statistics, Chicago, IL, USA) and Mplus (Version 8.6; Muthén & Muthén, Los Angeles, CA, USA) for Macintosh. Data are presented as mean ± standard error of the mean (SEM). Unless indicated differently, tests were two-tailed with the significance level set at a *p*-value of < 0.05. We a-priori calculated power-analyses using the statistical software G^*^Power for Macintosh (Version 3.1.9.6; Heinrich Heine University Düsseldorf, Germany). Based on previous research (Van der Klink et al., 2001; Hammerfald et al., 2006; Richardson and Rothstein, 2008; Limm et al., 2011; Kröll et al., 2017), we conservatively (1) expected small to medium sized group differences between SMT- and AT-groups (i.e., *f* = 0.15 in a 2 (group) × 3 (T) repeated measures ANOVA) and (2) presumed the lowest average correlation of repeated measures to be *r* = 0.40. To obtain a power of (1 – β) = 0.80, analyses have to be run on *N* = 88 participants. All data were tested for normal distribution and homogeneity of variance using Kolmogorov–Smirnov and Levene's tests. We applied Huynh–Feldt correction where appropriate. As not all assumptions were completely fulfilled and due to the number of missing data at follow-up assessments (see Figure 2 and Table 1), we conducted complementary robust analyses with Mplus. We used the robust full-information maximum-likelihood (FIML) estimator implemented in Mplus to estimate our models. In case of missing data, FIML performs well if the data are at least missing at random (MAR). Little's MCAR test (χ^2^(288) = 241.85, *p* = 0.98; 15 missing data patterns in the data) revealed that we may consider the data to be missing (completely) at random. If applicable, we report various model fit indices, including the Chi-Square Goodness-of-Fit test with a non-significant chi-square value indicating a good model fit, Root Mean Square Error of Approximation (RMSEA) with values below 0.05 indicating a good model fit, Comparative Fit Index (CFI) with values above 0.95 indicating a good model fit, and Standardized Root Mean Square Residual (SRMR) with values below 0.05 indicating a good model fit. Effect size parameters partial η^2^ ($\eta_{\text{p}}^{2}$; effect size conventions $\eta_{\text{p}}^{2}$: 0.01 = small; 0.06 = medium; 0.14 = large) and *d* (effect size conventions |*d|*: 0.20 = small; 0.50 = medium; 0.80 = large) are reported where appropriate.

**Table 1:** Group characteristics of the stress management training (SMT)-group and the alternative training (AT)-group at timepoint (T) 1, T2, and T3.

	**Group characteristics T1 (*****N*** = **108)**			**Group characteristics T2 (*****N*** = **86)**		**Group characteristics T3 (*****N*** = **66)**	
	**SMT-group *n* = 65**	**AT-group *n* = 43**	***p*-value**	**SMT-group *n* = 50**	**AT-group *n* = 36**	**SMT-group *n* = 36**	**AT-group *n* = 30**
Gender (*N*)	52 women 13 men	*n* = 42 26 women 16 men	**0.040**				
Age (years)	*n* = 59 32.88 ± 1.09 (20–54)	*n* = 42 31.02 ± 1.11 (19–49)	0.25				
ISBF-total-score	2.95 ± 0.07 (1.50–4.00)	3.02 ± 0.08 (2.07–4.14)	0.53	3.25 ± 0.06 (2.36–4.21)	2.88 ± 0.08 (1.86–3.57)	*n* = 35 3.45 ± 0.06(2.57–4.14)	2.87 ± 0.10 (1.71–3.64)
CogProb	2.92 ± 0.09 (1.00–4.60)	3.13 ± 0.09 (1.80–4.00)	0.13	3.22 ± 0.06 (2.20–4.20)	2.93 ± 0.10 (2.00–4.00)	*n* = 35 3.35 ± 0.08(2.00–4.40)	2.96 ± 0.12 (1.60–4.00)
AngExAs	3.21 ± 0.09 (1.33–5.00)	3.22 ± 0.09 (2.00–4.67)	0.96	3.33 ± 0.10 (1.33–5.00)	3.24 ± 0.10 (2.00–4.00)	*n* = 35 3.51 ± 0.08(2.67–5.00)	3.12 ± 0.11 (2.00–4.00)
SocRes	3.08 ± 0.13 (1.00–5.00)	3.43 ± 0.15 (2.00–5.00)	0.08	3.30 ± 0.15 (1.00–5.00)	3.18 ± 0.15 (2.00–5.00)	*n* = 35 3.39 ± 0.14 (1.00–5.00)	3.02 ± 0.17 (1.50–5.00)
RelaxAb	2.02 ± 0.11 (1.00–4.50)	2.05 ± 0.12 (1.00–4.00)	0.85	2.81 ± 0.12 (1.00–4.50)	2.04 ± 0.13 (1.00–4.00)	*n* = 35 3.01 ± 0.14 (1.00–5.00)	2.25 ± 0.18 (1.00–4.00)
PBodTens	3.43 ± 0.13 (1.00–5.00)	3.00 ± 0.16 (1.00–5.00)	**0.036**	3.59 ± 0.14 (1.00–5.00)	2.76 ± 0.15 (1.00–4.00)	*n* = 35 3.39 ± 0.15(1.00–5.00)	2.72 ± 0.15 (1.00–4.00)
Relaxation after work	12.95 ± 0.45 (4–20)	14.84 ± 0.43 (8– 20)	**0.005**	14.30 ± 0.46 (5 −20)	13.97 ± 0.48 (7–19)	*n* = 35 14.89 ± 0.56 (8–20)	13.17 ± 0.53 (7–20)
PSS	3.57 ± 0.07 (1.50– 4.00)	3.54 ± 0.07 (2.25–4.00)	0.74	3.65 ± 0.07 (1.50–4.00)	3.43 ± 0.09 (2.38–4.00)	*n* = 35 3.71 ± 0.08 (1.38–4.00)	3.22 ± 0.11 (2.00–4.00)
VE	*n* = 62 11.00 ± 0.59 (0–18)	7.23 ± 0.66 (0–17)	**< 0.001**	7.60 ± 0.62 (0–16)	8.97 ± 0.83 (0–18)	*n* = 35 7.46 ± 0.90(0–18)	8.20 ± 0.98 (0–17)
SRS-total-score	65.74 ± 0.98 (51–84)	54.51 ± 1.22 (38–68)	**< 0.001**	56.90 ± 1.26 (44–79)	56.61 ± 1.53 (39–78)	52.75 ± 1.18 (42–66)	58.17 ± 1.75 (42–78)
RWO	10.68 ± 0.30 (5–15)	8.70 ± 0.27 (5–11)	**< 0.001**	9.10 ± 0.31 (5–14)	8.75 ± 0.36 (5–12)	8.17 ± 0.37 (5–13)	9.30 ± 0.42 (5–13)
Resigned attitude toward one's job	11.43 ± 0.56 (4–21)	11.79 ± 0.69 (4–20)	0.68	10.14 ± 0.68 (4–19)	12.25 ± 0.69 (5–21)	*n* = 35 9.69 ± 0.72 (4–20)	13.07 ± 0.76 (5–21)
Trait anger	*n* = 64 20.28 ± 0.66 (10–37)	18.19 ± 0.59 (12– 31)	**0.028**	*n* = 49 17.90 ± 0.57 (10–27)	18.86 ± 0.69 (13–30)	*n* = 34 17.47 ± 0.73 (11–27)	19.30 ± 0.78 (13–29)
ERI ratio	*n* = 60 0.82 ± 0.04 (0.22–2.12)	*n* = 42 0.74 ± 0.04 (0.29–1.67)	0.20				
STAI	*n* = 58 39.52 ± 1.10 (24–63)	*n* = 35 38.43 ± 1.23 (24–54)	0.53				
ADS-K	*n* = 58 9.78 ± 0.91 (0–31)	*n* = 36 10.92 ± 1.05 (0–26)	0.42				

Values are means ± SEM (range) if not indicated differently.

ISBF-total-score, Inventory for Assessment of Stress Management Skills total-score; CogProb, ISBF subscale cognitive-strategies-and-problem-solving; AngExAs, ISBF subscale adequate-anger-expression-and-assertiveness; SocRes, ISBF subscale identification-and-use-of-social-resources, RelaxAb, ISBF subscale relaxation-abilities; PBodTens, ISBF subscale perception-of-bodily-tension; PSS, perceived social support; VE, vital exhaustion; SRS-total-score, Stress-Reactivity-Scale total-score; RWO, SRS subscale reactivity-to-work-overload; ERI ratio, effort reward imbalance ratio; STAI, state-trait-anxiety-inventory; ADS-K, German version of the “Center for Epidemiological Studies Depression Scale”; T1, timepoint 1; T2, timepoint 2; T3, timepoint 3; SMT-group, stress management training group; AT-group, alternative training group; n, sample size; deviating sample sizes of a parameter are indicated; *p*-values two-tailed *p*-values either of χ^2^-test (gender) or independent t-tests (all other variables).

Significant values are highlighted in bold (*p* < 0.05).

#### 2.4.1 Changes over time in the study groups

**Figure 3:**
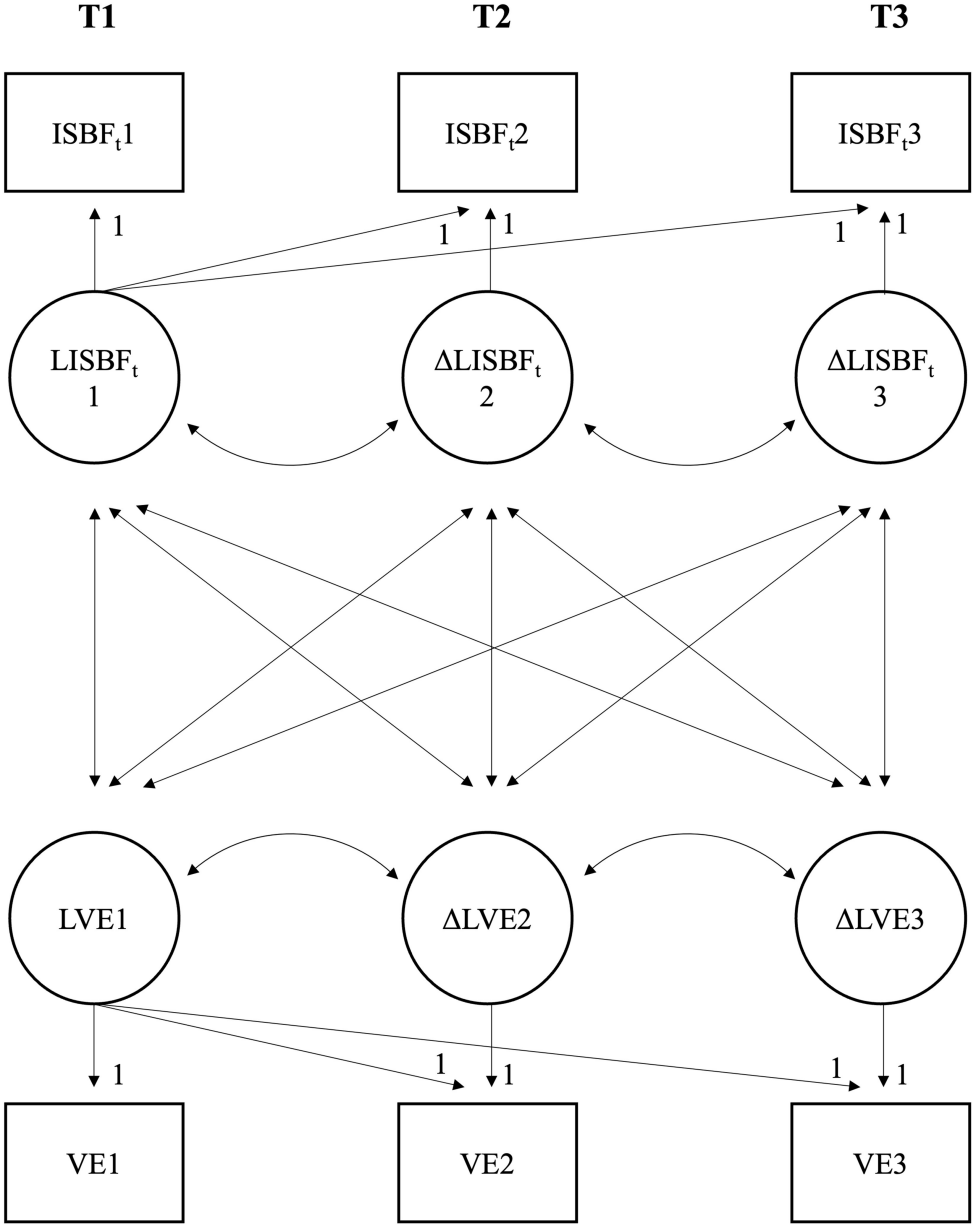
A simplified graphical representation of the main multigroup latent difference model (LD model) based on two (instead of seven) outcome variables, namely Inventory for Assessment of Stress Management Skills total-score (ISBF_t_) and vital exhaustion (VE). One baseline-change model is calculated for the stress management training (SMT)-group and simultaneously one for the alternative training (AT)-group (i.e., multigroup). Mean baseline values were calculated for timepoint (T) 1 (LISBF_T_1 and LVE1). Baseline-change scores were calculated for T2 and T3 (ΔLISBF_T_2 and ΔLVE2: T2-T1; ΔLISBF_T_3 and ΔLVE3: T3-T1).

To test whether the SMT induced improvements in stress-related (perceived mastery of functional stress management skills (ISBF-total-score and its subscales), relaxation after work, VE, stress reactivity (SRS-total-score and the subscale reactivity-to-workoverload)), work-related (job dissatisfaction), and specific-context-related (trait anger, PSS) outcome measures compared to the AT, we first conducted *repeated measures MANOVAs* in SPSS with group as an independent variable and main (i.e., without subscales) outcome measures (T1, T2, and T3) as dependent variables. Outcome variables with expected increases over time (i.e., ISBF-total-score, relaxation after work and PSS; see Figure 1) were recoded for MANOVAs that a decrease always represents an improvement. Because of missing data, we ran two repeated measures MANOVAs. In the first MANOVA, we excluded all participants with missing values (listwise deletion), while we used “last observation carried forward” (LOCF) in the second MANOVA. *Post-hoc* testing of a significant three-way (i.e., outcome variables-by-time-by-group) interaction effects comprised repeated measures ANOVAs for all outcome variables, including subscales. Given the baseline difference in gender between the groups but not in age, baseline work stress, anxiety, and depression (see Table 1), we conducted all analyses both without and with controlling for gender. Second, we estimated *multigroup latent difference models* (LD models; McArdle, 1988) in MPlus as complementary analyses, allowing us to perform analyses including all participants. More precisely, to model the actual change of our participants in response to SMT or ATs, we estimated baseline-change-models. In these models, increases and decreases at post (T2) or follow-up (T3) as compared to baseline (T1) are modeled as differences between latent factor scores separately for the SMT- and the AT-group (see Figure 3 for a simplified graphical representation). Thus, the LD model allows us to examine baseline scores and mean differential changes over time, i.e., from pre-to-post and pre-to-follow-up. These baseline scores and, most importantly, mean differential changes over time are tested based on two-tailed *p*-values of *z*-tests. Effect sizes *d* for these effects are calculated by dividing unstandardized change scores by the standard deviation of a variable at T1. *Post-hoc*, we used one-tailed, independent *t*-tests to compare means at baseline and mean changes over time (“pre-to-post” and “pre-to-follow-up”) between the SMT- and the AT-group. Thus, these models provide results similar to those of repeated measures (M)ANOVAs. However, important advantages are that the LD models are generally more robust, missings can be estimated using FIML, and they allow modeling of all kinds of changes, including non-linear changes. Notably, we specified two LD models: First, in the main LD model, we tested the differential trajectories for the seven main outcome variables. Second, in an additional LD model, we accordingly tested the differential trajectories for the five ISBF subscales and the SRS subscale reactivity-to-work-overload.

#### 2.4.2 Mediation analyses

**Figure 4:**
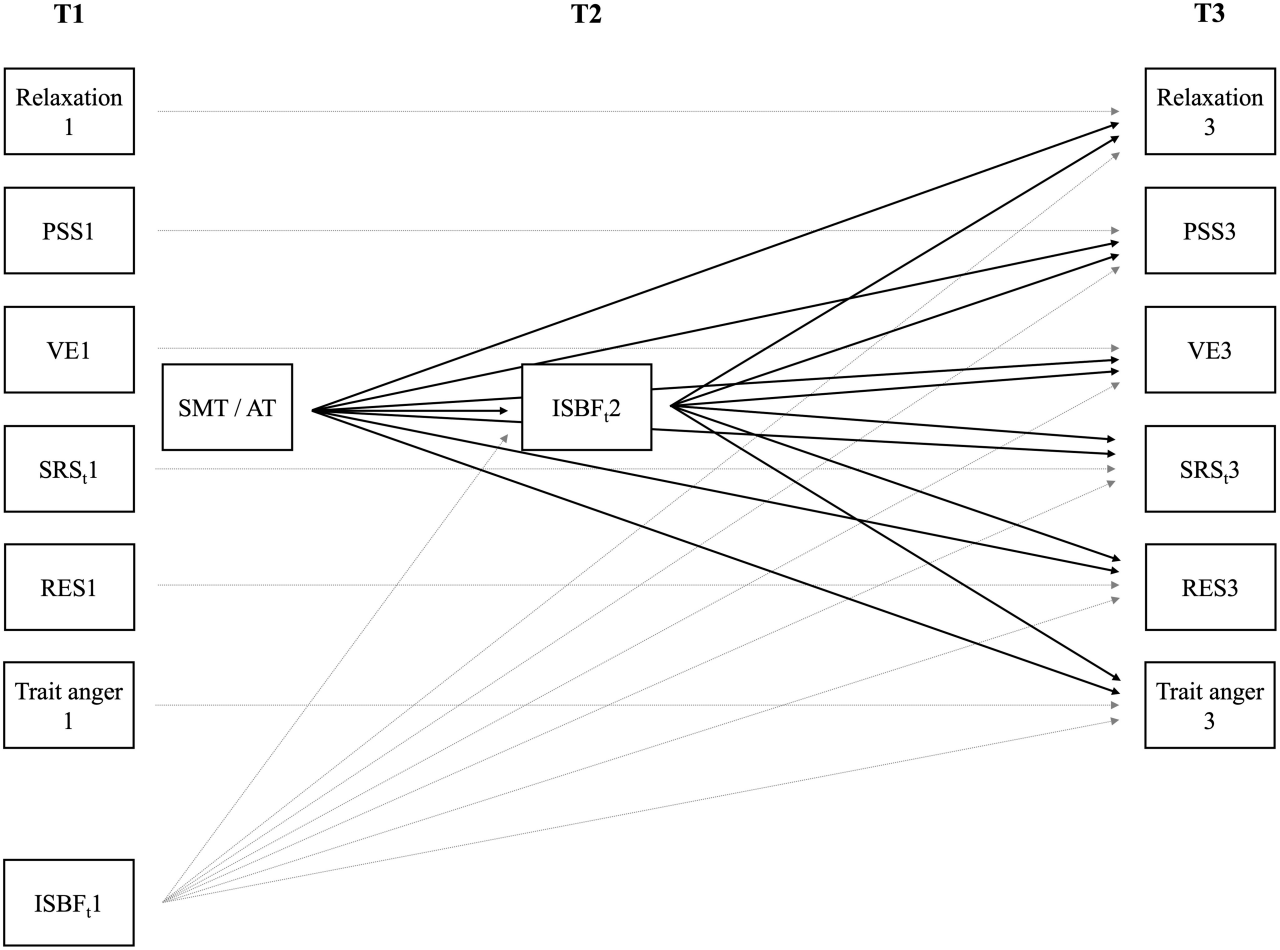
The main mediation model with Inventory for Assessment of Stress Management Skills total-score (ISBF_t_) at timepoint (T) 2 as mediator and the other main outcome variables at T3 as dependent variables (black lines) while controlling for their baseline (T1), i.e., before beginning of the stress management training (SMT) or the alternative training (AT) (gray lines). For reasons of clarity, we did not depict correlations across variables at T1 and at T3, and between T1 and group allocation. Relaxation, relaxation after work; PSS, perceived social support; VE, vital exhaustion; SRS_t_, Stress-Reactivity-Scale total-score; RES, resigned attitude toward one's job.

To test whether significant intervention-induced changes in perceived mastery of functional stress management skills would mediate significant intervention-induced changes in the other outcome variables, we ran multivariate mediation analyses with bootstrapping in Mplus. More specifically, we specified path models to examine whether the outcome variables at T3 can be predicted by SMT participation and whether this effect is mediated by changes in functional stress management skills (T2) while controlling for baseline scores. Thus, our mediation models allow us to consider the temporal precedence of variables influencing each other and thus to test for true mediation. In our main mediation model, we used the ISBF-total-score as mediator of the SMT effects on all other main outcomes (see Figure 4). In the additional mediation model, we used the SRS subscale reactivity-to-work-overload as the only dependent variable as it is statistically not possible to analyze SRS-total-score and its subscale reactivity-to-work-overload simultaneously as outcome variables in one path model. In *post-hoc* mediation models, we accordingly tested whether significant changes in ISBF subscales instead of the total-score would mediate training effects on other outcome variables. Notably, sample size varies slightly between mediation models because of missing data treatment in Mplus with the exclusion of participants with missing baseline values or a complete lack of post-training data (see Table 1) in such models. In all path models, statistical significance was evaluated based on one-sided 95% bootstrap confidence intervals (CI).

## 3 Results

### 3.1 Group characteristics

The total study sample consisted of 108 participants, with 65 participants in the SMT-group and 43 in the AT-group. The characteristics of the study groups at T1, T2, and T3 are detailed in Table 1. At T1, the SMT- and the AT-group did not significantly differ in age, ISBF-total-score, PSS, and job dissatisfaction (*p*'s ≥ 0.25). Moreover, there were no significant baseline group differences in work stress, anxiety, and depression between study groups (*p*'s ≥ 0.20). However, the SMT-group had lower scores in relaxation after work, higher scores in VE, SRS-total-score, and trait anger, as well as a higher proportion of women as compared to the AT-group (*p*'s ≤ 0.040).

### 3.2 Changes over time in the study groups

Repeated measures MANOVAs with group (SMT- vs. AT-group) as the independent variable and the seven main outcome measures as repeated (T1, T2, and T3) dependent variables with listwise deletion revealed a statistically significant three-way interaction between outcome variables, time, and group (*F*_(5.08,304.56)_ = 17.08, *p* < 0.001, $\eta_{\text{p}}^{2}$ = 0.22; with gender as covariate: *F*_(5.18,300.47)_ = 13.47, *p* < 0.001, $\eta_{\text{p}}^{2}$ = 0.19). Corresponding repeated measures MANOVAs with LOCF revealed similar results (three-way interaction: *F*_(4.56,464.56)_ = 19.15, *p* < 0.001, $\eta_{\text{p}}^{2}$ = 0.16; with gender as covariate: *F*_(4.64, 463.54)_ = 16.61, *p* < 0.001, $\eta_{\text{p}}^{2}$ = 0.14). The significant three-way interactions indicate differential trajectories of the main outcome variables depending on group, which we further explored *post-hoc*, again with missings excluded as well as LOCF, each without and with controlling for gender. Repeated measures ANOVAs for each main outcome variable separately revealed significant interactions of time-by-group with beneficial changes (i.e., increases in ISBF-total-score, relaxation after work, and PSS, and decreases in VE, SRS-total-score, job dissatisfaction, and trait anger) in the SMT-group compared to the AT-group for each of the seven outcome variables (*p'*s ≤ 0.010). Further subscale *post-hoc* testing revealed significant interactions of time-by-group for the ISBF subscales cognitive-strategies-and-problem-solving (*p'*s ≤ 0.002), identification-and-use-of-social-resources (*p'*s ≤ 0.037), relaxation-abilities (*p'*s ≤ 0.001), and for the SRS subscale reactivity-to-work-overload (*p'*s < 0.001), all with improvements in the SMT-group as compared to the AT-group. The ISBF subscale adequate-anger-expression-and-assertiveness was significant with LOCF, both without and with controlling for gender and with listwise deletion when controlling for gender (*p'*s ≤ 0.045). No significant interaction was found for the ISBF subscale perception-of-bodily-tension (*p'*s ≥ 0.11). Detailed results of *post-hoc* repeated measures ANOVAs are provided in Supplementary material 2, Table S1.

Complementary LD models further confirmed the obtained results. As results of the repeated measures (M)ANOVAs were independent of gender, this variable was not considered in LD models to reduce the complexity of the models. Notably, running alternative LD models on residuals controlled for gender did not change results (data not shown). Table 2 provides detailed results. With regard to the *main LD model*, we found the expected increases from “pre-to-post” and “pre-to-follow-up” for ISBF-total-score and relaxation after work (*p'*s ≤ 0.001) and the expected decreases from “pre-to-post” and “pre-to-follow-up” for VE, SRS-total-score, job dissatisfaction, and trait anger (*p'*s ≤ 0.048) in the SMT-group. For PSS, where both groups displayed high values at baseline, the increase from “pre-to-post” did not reach statistical significance (*p* = 0.063), whereas the increase from “pre-to-follow-up” assessment did (*p* = 0.001). Overall, effect sizes of change within the SMT-group are small (*d*'s ≤ |0.20|, except for *d*(T2-T1 ISBF-total-score) = 1.09, *d*(T3-T1 ISBF-total-score) = 1.40, and *d*(T3-T1 PSS) = 0.56). For the AT-group, we found either non-significant changes or statistically significant but non-beneficial changes over time, indicating either preservation of the status quo or deterioration. *Post-hoc* calculated group comparisons of change scores between SMT- and AT-group revealed that all comparisons became significant with beneficial changes in the SMT-group. With regard to the *additional LD model* with the six subscales of interest, we found the expected decreases in the SMT-group from “pre-to-post” and “pre-to-follow-up” assessment for the SRS subscale reactivity-to-work-overload (*p'*s < 0.001) and the expected increases from “pre-to-post” and/or “pre-to-follow-up” assessment for all ISBF subscales (*p'*s ≤ 0.004) except for the subscale perception-of-bodily-tension, which did not significantly change over time (*p'*s ≥ *0.1*7). In the AT-group, we again observed statistically non-significant changes or significant non-beneficial changes over time. *Post-hoc* calculated group comparisons of change scores revealed significant group differences in change scores from “pre-to-post” and/or “pre-to-follow-up” in all subscales of interest, again with beneficial effects in the SMT-group as compared to the control group.

**Table 2:** Unstandardized baseline values and change scores of the multigroup latent difference models (LD models) and *post-hoc* group comparison between the stress management training (SMT)-group and the alternative training (AT)-group.

	**Baseline (T1)**			**Change pre-to-post (T2-T1)**			**Change pre-to-follow-up (T3-T1)**		
	**SMT-group**	**AT-group**	**group comparison**	**SMT-group**	**AT-group**	**group comparison**	**SMT-group**	**AT-group**	**group comparison**
	***Mean***	***Mean***	***p*-value (effect size)**	***Mean change p*-value (effect size)**	***Mean change p*-value (effect size)**	***p*-value (effect size)**	***Mean change p*-value (effect size)**	***Mean change p*-value (effect size)**	***p*-value (effect size)**
ISBF-total-score^a^	2.95	3.02	0.26 (0.13)	**0.30** ** < 0.001 (1.09)**	**−0.13 0.010 (−0.48)**	**< 0.001 (−1.14)**	**0.39** ** < 0.001 (1.40)**	−0.14 0.064 (−0.49)	**< 0.001 (−1.05)**
CogProb^b^	2.92	3.13	0.055 (0.32)	**0.28** ** < 0.001 (0.53)**	**−0.170 0.032 (−0.51)**	**< 0.001 (−0.85)**	**0.40** ** < 0.001 (0.75)**	−0.13 0.14 (−0.40)	**< 0.001 (−0.86)**
AngExAs^b^	3.21	3.22	0.48 (0.01)	0.12 0.28 (0.22)	0.03 0.75 (0.08)	0.29 (−0.11)	**0.37** ** < 0.001 (0.71)**	−0.09 0.47 (−0.22)	**0.002 (−0.58)**
SocRes^b^	3.09	3.43	**0.038 (0.35)**	**0.260.004 (0.24)**	−0.15 0.27 (−0.16)	**0.007 (−0.49)**	0.18 0.13 (0.17)	**−0.38 0.005 (−0.42)**	**0.001 (−0.65)**
RelaxAb^b^	2.02	2.05	0.42 (0.04)	**0.84** ** < 0.001 (1.19)**	0.00 0.99 (0.00)	**< 0.001 (−1.11)**	**1.09** ** < 0.001 (1.55)**	0.23 0.12 (0.40)	**< 0.001 (−0.82)**
PBodTens^b^	3.43	3.00	**0.016 (−0.43)**	0.17 0.17 (0.16)	−0.16 0.20 (−0.16)	**0.031 (−0.37)**	0.11 0.43 (0.11)	−0.20 0.16 (−0.20)	0.064 (−0.30)
Relaxation after work^a^	12.95	14.84	**0.001 (0.60)**	**1.260.001 (0.10)**	−0.74 0.10 (−0.10)	**0.001 (−0.66)**	**1.75** ** < 0.001 (0.14)**	**−1.33 0.017 (−0.17)**	**< 0.001 (−0.87)**
PSS^a^	3.57	3.54	0.37 (−0.07)	0.08 0.063 (0.31)	−0.10 0.13 (−0.52)	**0.012 (−0.45)**	**0.15 0.001 (0.56)**	**−0.21 0.010 (−1.05)**	**< 0.001 (−0.76)**
VE^a^	10.95	7.23	**< 0.001 (−0.83)**	**−3.15** ** < 0.001 (−0.14)**	**2.03 0.001 (0.11)**	**< 0.001 (1.11)**	**−3.77** ** < 0.001 (−0.17)**	**1.62 0.040 (0.09)**	**< 0.001 (0.97)**
SRS-total-score^a^	65.74	54.51	**< 0.001 (−1.42)**	**−8.76** ** < 0.001 (−0.14)**	**3.16 0.018 (0.05)**	**< 0.001 (1.44)**	**−12.54** ** < 0.001 (−0.20)**	**4.51 0.025 (0.07)**	**< 0.001 (1.48)**
RWO^b^	10.68	8.70	**< 0.001 (−0.97)**	**−1.46** ** < 0.001 (−0.25)**	0.140.66 (0.04)	**< 0.001 (0.79)**	**−2.48** ** < 0.001 (−0.43)**	0.44 0.32 (0.14)	**< 0.001 (0.94)**
Resigned attitude toward one's job^a^	11.43	11.79	0.34 (0.08)	**−1.02 0.048 (−0.05)**	0.73 0.23 (0.04)	**0.015 (0.43)**	**−1.56 0.007 (−0.08)**	1.14 0.14 (0.06)	**0.003 (0.55)**
Trait anger^a^	20.35	18.19	**0.008 (−0.48)**	**−2.34** ** < 0.001 (−0.08)**	0.98 0.20 (0.07)	**< 0.001 (0.67)**	**−2.35 0.001 (−0.08)**	1.28 0.16 (0.09)	**0.001 (0.62)**

All results of the two multigroup latent difference models (LD models) are depicted in this table.

a Results of main LD model, i.e., with main outcome variables.

b Results of additional LD model, i.e., with subscales of interest.

ISBF-total-score, Inventory for Assessment of Stress Management Skills total-score; CogProb, ISBF subscale cognitive-strategies-and-problem-solving; AngExAs, ISBF subscale adequate-anger-expression-and-assertiveness; SocRes, ISBF subscale identification-and-use-of-social-resources, RelaxAb, ISBF subscale relaxation-abilities; PBodTens, ISBF subscale perception-of-bodily-tension; PSS, perceived social support; VE, vital exhaustion; SRS-total-score, Stress-Reactivity-Scale total-score; RWO, SRS subscale reactivity-to-work-overload; ERI ratio, effort reward imbalance ratio; T1, timepoint 1; T2, timepoint 2; T3, timepoint 3; SMT-group, stress management training group; AT-group, alternative training group; *p*-values group comparison, one-tailed *p*-values of *post-hoc* independent t-tests with effect size d; *p*-values mean change, two-tailed *p*-values of z-tests with effect sizes calculated by dividing unstandardized change score by the standard deviation of the variable at T1.

Significant values are highlighted in bold (*p* < 0.05).

### 3.3 Mediation analyses

For reasons of clarity, we present mediation results in a condensed way. For complete results of mediation analyses with ISBF-total-score as a mediator, see Table 3. For mediation analyses with ISBF subscales as mediators, see Supplementary material 2, Table S2 (model fit information) and Table S3 (model results). Statistical significance was evaluated based on one-sided 95% bootstrap CIs and only the relevant CI (lower (LL) or upper (UL)) limits for statistical significance are presented.

**Table 3:** Standardized estimates and bootstrapped 90% confidence intervals (CI) of the total, direct, and mediated (indirect) effects of the mediation models with ISBF-total-score as mediator.

	**Total effect**			**Direct effect of group (SMT-/AT-group)**			**Mediated indirect effect via ISBF-total-score T2**		
	**Estimate**	**Lower limit**	**Upper limit**	**Estimate**	**Lower limit**	**Upper limit**	**Estimate**	**Lower limit**	**Upper limit**
**Main mediation model (*****n*** = **84)**									
Relaxation after work T3	**0.46**	*0.310*	0.589	**0.31**	*0.081*	0.481	**0.15**	*0.033*	0.323
PSS T3	**0.31**	*0.176*	0.473	0.18	*−0.029*	0.392	0.13	*0.000*	0.304
VE T3	**−0.39**	−0.557	*−0.218*	−0.16	−0.355	*0.070*	**−0.24**	−0.401	*−0.116*
SRS-total-score T3	**−0.60**	−0.769	*−0.439*	**−0.36**	−0.606	*−0.116*	**−0.24**	−0.429	*−0.085*
Resigned attitude toward one's job T3	**−0.41**	−0.554	*−0.248*	**−0.30**	−0.532	*−0.097*	−0.10	−0.245	*0.064*
Trait anger T3	**−0.33**	−0.497	*−0.154*	−0.11	−0.363	*0.145*	**−0.22**	−0.419	*−0.045*
ISBF-total-score T2				**0.43**	*0.320*	0.530			
**Additional mediation model (*****n*** = **87)**									
RWO T3	**−0.39**	−0.572	*−0.220*	−0.18	−0.421	*0.058*	**−0.21**	−0.390	*−0.067*
ISBF-total-score T2				**0.42**	*0.310*	0.518			

N, 10,000 Bootstrapping resamples; n, sample size; T2, timepoint 2; T3, timepoint 3; PSS, perceived social support; VE, vital exhaustion; SRS-total-score, Stress-Reactivity-Scale total-score; ISBF-total-score, Inventory for Assessment of Stress Management Skills total-score; RWO, SRS subscale reactivity-to-work-overload; SMT-group, stress management training group; AT-group, alternative training group.

Statistical significance was evaluated based one-sided 95% bootstrap confidence intervals (CI), the relevant CI limits for statistical significance are highlighted in italics; significant estimates based on CIs are highlighted in bold (*p* < 0.05).

With regard to the *ISBF-total-score as mediator*, the main mediation model (see Figure 4; χ^2^(36) = 39.22, *p* = 0.33; RMSEA = 0.03 (90% CI = [0.000; 0.087]); CFI = 0.99; SRMR = 0.08) and the additional mediation model (χ^2^(1) = 0.09, *p* = 0.76; RMSEA = 0.00 (90%CI = [0.000; 0.192]); CFI = 1.00; SRMR = 0.01) fit the data well. In the main mediation model, we found the total effects of group, i.e., training, on all outcome variables (95% CIs for expected increases in SMT- vs. AT-group = [LLs ≥ 0.176]; 95%CIs for expected decreases in SMT- vs. AT-group = [ULs ≤ −0.154]). These total effects decompose into a mediated, i.e., indirect effect (reflecting the specified training effects via ISBF-total-score T2) and a direct effect of group, i.e., training effect on the outcome variable (reflecting unspecific training effects independent of ISBF-total-score changes). We found indirect effects supporting the expected mediation model via ISBF-total-score for relaxation after work (95% CI = [LL = 0.033]), VE (95% CI = [UL = −0.116]), SRS-total-score (95% CI = [UL = −0.085]), and trait anger (95% CI = [UL = −0.045]). Furthermore, we observed direct effects for relaxation after work (95% CI = [LL = 0.081]), SRS-total-score (95% CI = [UL = −0.116]), and job dissatisfaction (95% CI = [UL = −0.097]), but not for PSS, VE, and trait anger. The significant direct effect of group, i.e., training, on ISBF-total-score T2 (95% CI = [LL = 0.320]) reflects training-induced increases in functional stress management skills. In the additional mediation model with the SRS subscale reactivity-to-work-overload as outcome variable, we also found a statistically significant total effect (95% CI = [UL = −0.220]) with a statistically significant indirect (95% CI = [UL = −0.067]), but no direct effect. The direct effect of group, i.e., training, on ISBF-total-score T2 (95% CI = [LL = 0.310]) was again significant.

With respect to mediation analyses with the *ISBF subscales as mediators*, the subscale cognitive-strategies-and-problem-solving proved to be the most important mediator since we found indirect effects via this subscale for all outcomes (main model and additional model: 95% CIs for expected increases in the SMT- vs. AT-group = [LLs ≥ 0.004]; 95% CIs for expected decreases in the SMT- vs. AT-groups = [ULs ≤ −0.078]), except job dissatisfaction. In addition, the subscale identification-and-use-of-social-resources had indirect effects on VE and the SRS subscale reactivity-to-work-overload (95% CIs = [ULs ≤ −0.002]). For the ISBF subscales adequate-anger-expression-and-assertiveness and relaxation-abilities, there were no or fewer indirect effects. Mediation analyses with the ISBF subscales as mediators are described in more detail in Supplementary material 3. Taken together, over all mediation analyses, we found indirect effects via ISBF-total-score and/or subscales for all outcome variables except for job dissatisfaction, supporting our hypothesis that SMT effects are mediated via functional stress management skills and, in particular, via cognitive-strategies-and-problem-solving, i.e., perceived mastery of changing cognition.

## 4 Discussion

In this study, we investigated the effects of an occupational cognitive-behavioral SMT compared to ATs on different outcome measures and examined mediating effects of perceived mastery of functional stress management skills, in particular, of changing cognitions, over a period of 3 months.

We first found the expected beneficial *effects of the SMT* on stress-related, work-related, and specific-context-related measures. More specifically, regarding stress-related measures, we observed improvements in terms of increases in perceived mastery of functional stress management skills in total (ISBF-total-score), including improvements in the subscales cognitive-strategies-and-problem-solving, adequate-anger-expression-and-assertiveness, identification-and-use-of-social-resources, and relaxation-abilities. Moreover, we found improvements in terms of increases in relaxation after work and decreases in stress reactivity (in particular, to work overload) and exhaustion. We also found decreases in the work-related measure of job dissatisfaction. With respect to specific-context-related measures, social support increased and anger decreased. Overall, our findings are in line with the results of previous meta-analyses showing beneficial effects of cognitive-behavioral SMTs, especially with regard to psychological measures (Saunders et al., 1996; Van der Klink et al., 2001; Richardson and Rothstein, 2008; Kröll et al., 2017). In more detail, beneficial effects of cognitive-behavioral SMTs have previously been shown for comparable stress-related (e.g., recovery experiences and thus relaxation (Siu et al., 2014), stress reactivity (Limm et al., 2011), exhaustion (Higgins, 1986; Norvell et al., 1987; Kushnir and Malkinson, 1993; Van Rhenen et al., 2005; Brinkborg et al., 2011; Lloyd et al., 2013; Riley et al., 2017; Ojala et al., 2019)), work-related (e.g., job (dis)satisfaction (Forman, 1981; Cecil and Forman, 1990; Bunce and West, 1996; Maddi et al., 1998; Nickel et al., 2007)), and specific-context-related (e.g., anger (Keyes and Dean, 1988; Nickel et al., 2007), social support (Freedy and Hobfoll, 1994; Maddi et al., 1998)) measures. Notably, these beneficial effects have not been observed unequivocally (Grønningæter et al., 1992; Freedy and Hobfoll, 1994; Bond and Bunce, 2000; de Jong and Emmelkamp, 2000; Munz et al., 2001; Willert et al., 2009; Siu et al., 2014). With respect to cognitive-behavioral SMT effects on stress management skills, our results are in line with findings of non-clinical studies assessing the frequency of use of coping (Long, 1988; de Jong and Emmelkamp, 2000; Bekker et al., 2001; Zołnierczyk-Zreda, 2002; Gardiner et al., 2004; Willert et al., 2009; Alkhawaldeh et al., 2020) and with findings of clinical studies (Penedo et al., 2004, 2006; Antoni et al., 2006; Jensen et al., 2013; Gudenkauf et al., 2015; Marsland et al., 2020) and one non-clinical study (Riley et al., 2017) assessing perceived mastery of stress management skills. Notably, these studies found improvements in coping or stress management skills on at least one scale. Overall, most studies report improvements in some outcome measures, and to the best of our knowledge, only a few studies report a similar broad range of beneficial SMT effects.

In the present SMT, participants were trained in cognitive restructuring, self-instructions, systematic problem-solving, and relaxation as *basic stress reduction techniques*. As outlined in the introduction and depicted in Figure 1, we consider the reported increases in the ISBF subscales cognitive-strategies-and-problem-solving and relaxation-abilities as well as in the relaxation after work scale as a direct result of successful training. Moreover, as we explicitly trained the basic techniques in the contexts of anger and social support, we further interpreted the observed improvements in the ISBF subscales, adequate-anger-expression-and-assertiveness and identification-and-use-of-social-resources, as well as in PSS and trait anger to result from successful technique acquisition and mastery in the respective contexts. In contrast, as there were no specific parts of the training addressing exhaustion, stress reactivity, or job dissatisfaction, the observed beneficial training effects in these outcomes more likely result from the total training (see Figure 1). There were no SMT-induced improvements in the ISBF subscale perception-of-bodily-tension, a skill that notably was not explicitly trained.

Second, regarding the *mechanisms underlying the observed SMT effects* on outcome variables other than ISBF scales, our mediation analyses confirmed mediating effects of perceived mastery of functional stress management skills in general and specifically of the subscale cognitive-strategies-and-problem-solving; these results apply to all outcome variables except job dissatisfaction. This suggests that the effectiveness of our occupational cognitive-behavioral SMT can specifically be attributed to improvements in perceived mastery of functional stress management skills. In this regard, our results provide empirical evidence in support of the framework of moderators, mediators, and mechanisms of change of occupational SMTs proposed by Bunce (1997), where changes in terms of mastery of coping are relevant to induce beneficial SMT outcomes. In more detail, the ISBF-total-score emerged as a mediator of change for almost all outcomes (except for job dissatisfaction and social support). This is in line with the finding that *active training* of coping skills is of principal importance for beneficial SMT effects (West et al., 1984) and, for the first time, extends results from clinical studies in the context of cancer (Penedo et al., 2004, 2006; Marsland et al., 2020) to a non-clinical occupational context. Moreover, as expected, based on the transactional model of stress (Lazarus and Folkman, 1984), we found the ISBF subscale cognitive-strategies-and-problem-solving to mediate training effects on almost all outcome variables, with job dissatisfaction being the only exception. This suggests that perceived mastery of changing cognitions seems to be the most important stress management skill, corroborating findings on this subscale in mothers of children newly diagnosed with cancer (Marsland et al., 2020). With regard to the non-clinical context, this finding further supports and extends the findings that beneficial changes in dysfunctional cognition, stress appraisal, or psychological flexibility mediate or at least relate to improvements after SMT (Bond and Bunce, 2000; Gaab et al., 2003; Hammerfald et al., 2006; Keogh et al., 2006; Flaxman and Bond, 2010; Leung et al., 2010; Brinkborg et al., 2011; Lloyd et al., 2013).

In addition, we observed the subscale identification-and-use-of-social-resources to mediate training effects on exhaustion and the SRS subscale reactivity-to-work-overload. This finding can be interpreted in the context of the social support-reactivity hypothesis where social support is supposed to buffer stress effects with resulting beneficial effects on health (Lepore, 1998; Christenfeld and Gerin, 2000). Mediation results for the ISBF subscales adequate-anger-expression-and-assertiveness and relaxation-abilities are discussed in Supplementary material 4. Interestingly, the observed improvements in job dissatisfaction were not mediated by SMT-induced improvements in functional stress management skills. This suggests that other mechanisms have to account for the improvement in job dissatisfaction. We can only speculate that general training effects may play a role. However, it is also conceivable that the offer to undergo a SMT without financial costs during work-time at full payment is perceived as appreciation and concern by a responsible employer that cares for employees. At the same time, the finding of non-mediation by stress management skills in general and cognitive strategies, in particular, also suggests that there are limits that even optimal individual coping cannot overcome. It needs to be considered that a stressful environment is capable of inducing stress in probably everyone, if the level of stress induction is high enough, particularly, if the level of control or reward is low (e.g., Karasek, 1979; Siegrist, 1996). Therefore, the responsibility for health and wellbeing should not be left only at the individual employee level. Instead, it should be considered an explicit responsibility of employers to establish a workplace compatible for health and wellbeing of employees. Overall, achieving the best effects for a healthy workplace requires responsibility not only at the level of the individual but also of the work group, leader, and organization (Nielsen and Christensen, 2021). Notably, cognitive-behavioral SMTs are promising for contributing to a healthy workplace at the individual level. However, they are unlikely to compensate for substantial workplace deficits that require activity at further levels.

*Clinical implications* of our study include that cognitive-behavioral SMTs seem to be an effective way to teach employees techniques to reduce stress and the resulting burden that are primarily mediated by improvements in perceived mastery of changing cognitions. Therefore, SMTs should particularly pay attention to sufficiently teach and actively train and practice techniques such as cognitive restructuring, self-instructions, and systematic problem-solving. Further implications are that the group-setting of medium group size (7–12 participants) and a combination of teaching basic stress reduction techniques and training them in specific stress-related contexts seem well-suited for successful training effects. Given that other cognitive-behavioral SMTs have already been shown to have long-lasting beneficial effects over several years (Li et al., 2017; Herr et al., 2018), it is conceivable that our results are of similar persistence.

*Strengths* of our study include the comparison of our SMT-group with the AT-group to account for unspecific placebo effects of interventions in general. Second, the SMT was carried out according to a protocol based on well-established stress reduction programs (Kaluza, 1996; Siegrist and Silberhorn, 1998; Reschke and Schröder, 2000; Wiegard et al., 2000; Wagner-Link, 2001; Meichenbaum, 2017). Third, our study design with post and follow-up assessment allowed for considering the temporal precedence of variables influencing each other and thus to test for a true mediation. Finally, we used different and, in particular, state-of-the-art statistical methods to deal with missings, including listwise deletion as well as LOCF and FIML, following the intention-to-treat-approach, that, notably, all provided comparable results. *Limitations* of our study include the relatively low sample size, in particular, regarding the return rate of the follow-up assessment questionnaires (see Figure 2) that we, however, compensated for statistically. Second, we could not use a randomized group allocation as the company offered participation in the different group trainings as a personnel development procedure on a voluntary basis. Third, the SMT-group had less favorable baseline levels in some outcome measures (relaxation after work, VE, stress reactivity, and trait anger) at baseline than the AT-group, suggesting that the SMT-group was more stressed at study entry, which may have influenced the potential for improvements. We interpret these baseline differences to result from the voluntary, non-randomized group assignment. In addition, the SMT-group comprised a higher proportion of women as compared to the AT-group, which however did not affect our results. Given this, further studies should include a randomized group assignment to overcome these limitations of our field study.

Taken together, our findings provide further evidence that occupational cognitive-behavioral SMTs are effective in reducing stress experience and the resulting burden as indicated by beneficial effects of our SMT on our outcome measures. Moreover, the beneficial effects of cognitive-behavioral SMTs seem to be mediated by SMT-induced improvements in perceived mastery of functional stress management skills, in particular, of changing cognitions. Future studies are needed to confirm these results in larger samples with randomized group allocation and to determine whether results can be generalized to other occupational sectors than health insurance and different working conditions. Moreover, it should be examined how such interventions on an individual level could be combined with interventions addressing demands and resources at a group, leader, and organizational level to achieve the best effects (Nielsen and Christensen, 2021).

## Data availability statement

The raw data supporting the conclusions of this article will be made available by the corresponding author, without undue reservation.

## Ethics statement

Ethical review and approval was not required for the study on human participants in accordance with the local legislation and institutional requirements. The participants provided their written informed consent to participate in this study.

## Author contributions

Conception and design of the study, investigation, funding acquisition, and supervision: PHW. Data curation and drafting the manuscript: PHW and AA. Analysis of the data and visualization: AA, PHW, and FWN. Interpretation of the data: PHW, AA, FWN, UE, and NKS. Critical revision of the manuscript: FWN, NKS, and UE. All authors approved the final version to be published and agree on being accountable for all aspects of the work.
