# Supplementary material for: Beneficial effects of a cognitive-behavioral occupational stress management group training: the mediating role of changing cognitions
Source: Front Psychol. 2024 Jul 29;14:1232172. doi: 10.3389/fpsyg.2023.1232172 (PMC11318457; doi:10.3389/fpsyg.2023.1232172)
Supplement: Supplementary file 1 [file Presentation_1.pdf]

Supplementary Material

Beneficial effects of a cognitive-behavioral occupational stress management group training: the mediating role of changing cognitions

Petra H. Wirtz, Alisa Auer, Norbert K. Semmer, Ulrike Ehlert, Fridtjof W. Nussbeck

1 Supplementary Material 1: Previous publication of data of the study

Parts of this study were used for psychometric validation of the ISBF (Wirtz et al., 2013). More precisely, within the employee sample 3 of that publication, we used the baseline assessment of the questionnaires ISBF (total-score and all subscales), SRS total-score, BSSS subscale PSS, and VE of all participants to calculate the factor structure and internal consistency of the ISBF scales as well its convergent validity with the three other scales. Moreover, preliminary data of the study were used for a doctoral thesis assessing SMT effects, including 30 participants of the experimental SMT-group and 20 participants of the AT-group (Stein, 2007). In that thesis, the effects of the SMT as compared to the AT were tested on most outcome variables except for the main questionnaire of interest of the present study, namely the ISBF-total-score and its subscales, and except for the SRS-total-score and its subscale stress-reactivity-to-work-overload.

2 Supplementary Material 2: Tables

Table S1

Results of post-hoc 2 (group) x 3 (timepoints) repeated measures ANOVAs without and with the covariate gender

	Listwise deletion<br>without covariate: *N*=62 (*n*=32 SMT-group; *n*=30 AT-group)<br>*(with covariate: N=61 (n=32 SMT-group; n=29 AT-group))*				Last observation carried forward (LOCF)<br>without covariate: *N*=104 (*n*=61 SMT-group; *n*=43 AT-group)<br>*(with covariate: N=103 (n=61 SMT-group; n=42 AT-group))*			
	$[df_{Num}, df_{Den}]$	*F*	*p*	η^2_p	$[df_{Num}, df_{Den}]$	*F*	*p*	η^2_p
ISBF-total-score	[1.57, 94.15] *(1.60, 92.96)*	11.63 *(7.54)*	**<.001** ***(.002)***	.16 *(.12)*	[1.41, 143.54] *(1.42, 142.28)*	19.33 *(16.17)*	**<.001** ***(<.001)***	.16 *(.14)*
CogProb	[1.67, 100.16] *(1.66, 93.39)*	10.51 *(7.69)*	**<.001** ***(.002)***	.15 *(.12)*	[1.46, 148.98] *(1.46, 146.29)*	14.76 *(12.42)*	**<.001** ***(<.001)***	.13 *(.11)*
AngExAs	[1.77, 106.11] *(1.80, 104.17)*	2.84 *(3.34)*	.069 ***(.044)***	.05 *(.05)*	[1.60, 163.57] *(1.63, 162.63)*	3.79 *(3.41)*	**.033** ***(.045)***	.04 *(.03)*
SocRes	[1.82, 109.04] *(1.85, 107.43)*	4.61 *(3.50)*	**.014** ***(.037)***	.07 *(.06)*	[1.69, 172.59] *(1.71, 171.10)*	7.07 *(6.57)*	**.002** ***(.003)***	.07 *(.06)*
RelaxAb	[2, 120] *(2, 116)*	10.53 *(7.43)*	**<.001** ***(<.001)***	.15 *(.11)*	[1.75, 178.36] *(1.77, 177.25)*	12.34 *(10.18)*	**<.001** ***(<.001)***	.11 *(.09)*
PBodTens	[1.77, 106.25] *(1.79, 104.07)*	.21 *(.06)*	.79 *(.93)*		[1.51, 154.22] *(1.52, 152.06)*	2.34 *(2.19)*	.11 *(.13)*	
Relaxation after work	[1.68, 100.69] *(1.67, 96.95)*	17.08 *(14.63)*	**<.001** ***(<.001)***	.22 *(.20)*	[1.50, 152.79] *(1.49, 148.61)*	16.86 *(13.94)*	**<.001** ***(<.001)***	.14 *(.12)*
PSS	[2, 120] *(2, 116)*	5.28 *(5.09)*	**.006** ***(.008)***	.08 *(.08)*	[1.85, 188.64] *(1.87, 186.71)*	8.30 *(7.97)*	**<.001** ***(<.001)***	.08 *(.07)*
VE	[2, 120] *(2, 116)*	15.13 *(12.31)*	**<.001** ***(<.001)***	.20 *(.18)*	[1.83, 186.52] *(1.85, 185.39)*	19.72 *(16.03)*	**<.001** ***(<.001)***	.16 *(.14)*
SRS-total-score	[1.86, 111.62] *(1.89, 109.65)*	33.07 *(25.82)*	**<.001** ***(<.001)***	.36 *(.31)*	[1.70, 173.66] *(1.72, 172.29)*	36.20 *(31.54)*	**<.001** ***(<.001)***	.26 *(.24)*
RWO	[1.74, 104.55] *(1.79, 103.96)*	15.87 *(13.52)*	**<.001** ***(<.001)***	.21 *(.19)*	[1.67, 169.94] *(1.70, 170.22)*	14.47 *(12.90)*	**<.001** ***(<.001)***	.12 *(.11)*

Resigned attitude toward one's job	[1.81, 108.37] *(1.84, 106.92)*	7.98 *(5.51)*	**<.001 *(.007)***	.12 *(.09)*	[1.66, 169.12] *(1.68, 167.70)*	8.33 *(7.07)*	**<.001 *(.002)***	.08 *(.07)*
Trait anger	[1.76, 105.64] *(1.77, 102.68)*	6.44 *(5.16)*	**.003 *(.010)***	.10 *(.08)*	[1.63, 166.08] *(1.63, 163.29)*	9.05 *(7.37)*	**<.001 *(.002)***	.08 *(.07)*

Note. Group-by-time interactions of post-hoc repeated measures ANOVAs without covariate and with covariate gender in parentheses and italics; ISBF-total-score=Inventory for Assessment of Stress Management Skills total-score; CogProb=ISBF subscale cognitive-strategies-and-problem-solving; AngExAs=ISBF subscale adequate-anger-expression-and-assertiveness; SocRes=ISBF subscale identification-and-use-of-social-resources, RelaxAb=ISBF subscale relaxation-abilities; PBodTens=ISBF subscale perception-of-bodily-tension; PSS=perceived social support; VE=vital exhaustion; SRS-total-score=Stress-Reactivity-Scale total-score; RWO=SRS subscale reactivity-to-work-overload; *n*=sample size; SMT-group=stress management training group; AT-group=alternative training group; df_{Num}=degrees of freedom numerator; df_{Den}=degrees of freedom denominator; significant values are highlighted in bold (p<.05).

Table S2

Model fit information of mediation models with ISBF subscales as mediator

	χ^2	RMSEA	CFI	SRMR
Main mediation models (*n*=84)				
via subscale CogProb T2	$\chi^2(36) = 46.37$, p=.12	.06 (90%CI = [.000;.103])	.97	.08
via subscale AngExAs T2	$\chi^2(36) = 41.49$, p=.24	.04 (90%CI = [.000;.092])	.98	.09
via subscale SocRes T2	$\chi^2(36) = 37.16$, p=.42	.02 (90%CI = [.000;.081])	1.00	.08
via subscale RelaxAb T2	$\chi^2(36) = 45.85$, p=.13	.06 (90%CI = [.000;.102])	.97	.08
Additional mediation models (*n*=87)				
via subscale CogProb T2	$\chi^2(1) = 0.84$, p=.36	.00 (90%CI = [.000;.274])	1.00	.02
via subscale AngExAs T2	$\chi^2(1) = 2.87$, p=.09	.15 (90%CI = [.000;.358)	.94	.05
via subscale SocRes T2	$\chi^2(1) = 1.33$, p=.25	.06 (90%CI = [.000;.300])	1.00	.03
via subscale RelaxAb T2	$\chi^2(1) = 0.65$, p=.42	.00 (90%CI = [.000;.262])	1.00	.02

Note. Main mediation models = SRS-total-score and other main outcome variables as dependent variables; additional mediation models = SRS subscale reactivity-to-work-overload as only dependent variable; *n*=sample size; T2=timepoint 2; CogProb=Inventory for Assessment of Stress Management Skills (ISBF) subscale cognitive-strategies-and-problem-solving; AngExAs=ISBF subscale adequate-anger-expression-and-assertiveness; SocRes=ISBF subscale identification-and-use-of social-resources, RelaxAb=ISBF subscale relaxation-abilities; RWO= Stress-Reactivity-Scale (SRS) subscale reactivity-to-work-overload; χ^2= Chi-Square Test of Model Fit; RMSEA=Root Mean Square Error Of Approximation; CFI=Comparative Fit Index; SRMR=Standardized Root Mean Square Residual; CI= confidence interval.

Table S3

Standardized estimates and bootstrapped 90% confidence intervals (CI) of the total, direct, and mediated (indirect) effects of mediation models with ISBF subscales as mediators

	Total effect			Direct effect of group (SMT- /AT-group)			Mediated indirect effect via subscale CogProb T2		
	Estimate	Lower Limit	Upper Limit	Estimate	Lower Limit	Upper Limit	Estimate	Lower Limit	Upper Limit
Main mediation model (*n*=84)									
Relaxation after work T3	**0.45**	*0.301*	0.586	**0.36**	*0.180*	0.508	**0.09**	*0.004*	0.205
PSS T3	**0.30**	*0.162*	0.458	0.11	*-0.015*	0.273	**0.19**	*0.107*	0.279
VE T3	**-0.36**	-0.527	*-0.175*	-0.16	-0.363	*0.061*	**-0.20**	-0.324	*-0.098*
SRS-total-score T3	**-0.54**	-0.704	*-0.380*	**-0.24**	-0.415	*-0.070*	**-0.30**	-0.428	*-0.186*
Resigned attitude toward one's job T3	**-0.40**	-0.561	*-0.226*	**-0.36**	-0.544	*-0.160*	-0.04	-0.159	*0.063*
Trait anger T3	**-0.30**	-0.477	*-0.120*	-0.12	-0.310	*0.089*	**-0.19**	-0.320	*-0.078*
CogProb T2				**0.37**	*0.241*	0.487			
Additional mediation model (*n*=87)									
RWO T3	**-0.36**	-0.540	*-0.176*	-0.12	-0.324	*0.086*	**-0.24**	-0.356	*-0.137*
CogProb T2				**0.36**	*0.237*	0.482			
							Mediated indirect effect via subscale AngExAs T2		
Main mediation model (*n*=84)									

Relaxation after work T3	**0.47**	*0.327*	0.601	**0.46**	*0.305*	0.589	0.02	*-0.012*	0.067
PSS T3	**0.32**	*0.189*	0.471	**0.30**	*0.164*	0.450	0.02	*-0.009*	0.071
VE T3	**-0.41**	-0.575	*-0.232*	**-0.38**	-0.536	*-0.201*	-0.04	-0.096	*0.015*
SRS-total-score T3	**-0.60**	-0.760	*-0.435*	**-0.56**	-0.739	*-0.395*	-0.03	-0.094	*0.013*
Resigned attitude toward one's job T3	**-0.42**	-0.560	*-0.262*	**-0.39**	-0.538	*-0.239*	-0.03	-0.074	*0.012*
Trait anger T3	**-0.32**	-0.493	*-0.136*	**-0.31**	-0.480	*-0.110*	-0.02	-0.077	*0.012*
AngExAs T2				0.11	*-0.044*	0.283			
Additional mediation model (*n*=87)									
RWO T3	**-0.36**	-0.547	*-0.161*	**-0.33**	-0.524	*-0.137*	-0.02	-0.084	*0.023*
AngExAs T2				0.08	*-0.077*	0.244			
							Mediated indirect effect via subscale SocRes T2		
Main mediation model (*n*=84)									
Relaxation after work T3	**0.46**	*0.310*	0.591	**0.44**	*0.279*	0.571	0.02	*-0.004*	0.077
PSS T3	**0.31**	*0.175*	0.472	**0.31**	*0.157*	0.469	0.00	*-0.026*	0.068
VE T3	**-0.40**	-0.569	*-0.214*	**-0.35**	-0.520	*-0.162*	**-0.05**	-0.124	*-0.004*
SRS-total-score T3	**-0.59**	-0.747	*-0.432*	**-0.55**	-0.708	*-0.372*	-0.04	-0.129	*0.003*
Resigned attitude toward one's job T3	**-0.39**	-0.547	*-0.222*	**-0.35**	-0.528	*-0.182*	-0.04	-0.084	*0.012*
Trait anger T3	**-0.31**	-0.484	*-0.129*	**-0.25**	-0.433	*-0.069*	-0.06	-0.144	*0.001*
SocRes T2				**0.16**	*0.028*	0.293			

Additional mediation model (*n*=87)									
RWO T3	**-0.40**	-0.579	*-0.226*	**-0.35**	-0.527	*-0.156*	**-0.05**	-0.153	*-0.002*
SocRes T2				**0.16**	*0.029*	0.290			
							Mediated indirect effect via subscale RelaxAb T2		
Main mediation model (*n*=84)									
Relaxation after work T3	**0.45**	*0.288*	0.591	**0.41**	*0.198*	0.598	0.04	*-0.059*	0.149
PSS T3	**0.29**	*0.158*	0.457	**0.43**	*0.273*	0.604	**-0.14**	*-0.213*	-0.059
VE T3	**-0.37**	-0.537	*-0.188*	**-0.40**	-0.608	*-0.197*	0.04	-0.068	*0.161*
SRS-total-score T3	**-0.59**	-0.744	*-0.441*	**-0.67**	-0.864	*-0.488*	0.08	-0.030	*0.200*
Resigned attitude toward one's job T3	**-0.39**	-0.553	*-0.229*	**-0.41**	-0.680	*-0.153*	0.02	-0.114	*0.175*
Trait anger T3	**-0.30**	-0.492	*-0.123*	-0.26	-0.533	*0.018*	-0.05	-0.203	*0.093*
RelaxAb T2				**0.44**	*0.312*	0.562			
Additional mediation model (*n*=87)									
RWO T3	**-0.40**	-0.583	*-0.223*	**-0.51**	-0.715	*-0.293*	0.11	-0.027	*0.216*
RelaxAb T2				**0.45**	*0.318*	0.568			

Note. *N*=10,000 Bootstrapping resamples; *n*=sample size; T2=timepoint 2; T3=timepoint 3; PSS=perceived social support; VE=vital exhaustion; SRS-total-score=Stress-Reactivity-Scale total-score; RWO=SRS subscale reactivity-to-work-overload; CogProb=Inventory for Assessment of Stress Management Skills (ISBF) subscale cognitive-strategies-and-problem-solving; AngExAs=ISBF subscale adequate-anger-expression-and-assertiveness; SocRes=ISBF subscale identification-and-use-of-social-resources, RelaxAb=ISBF subscale relaxation-abilities; SMT-group=stress management training group; AT-group=alternative training group; statistical significance was evaluated based on one-sided 95% bootstrap confidence intervals (CI), the relevant CI limits for statistical significance are highlighted in italics; significant estimates based on CIs are highlighted in bold ($p<.05$).

3 Supplementary Material 3: Results of mediation analyses with the ISBF subscales as mediators

Due to the sample size, we had to examine the *ISBF subscales as mediators* in separate path analytic models. For each ISBF subscales, that significantly changed over time, i.e. cognitive-strategies-and-problem-solving, adequate-anger-expression-and-assertiveness, identification-and-use-of-social-resources, and relaxation-abilities, we again specified two models, i.e., a main mediation model and an additional mediation model, resulting in a total of 8 models. All 8 models fit the data (acceptably) well (main models: χ^2's(36)≤46.37, *p's*≥.12; RMSEAs≤.06 (90%CIs=[LLs=.000;ULs≤.103]); CFIs≥.97; SRMRs≤.09 and additional models: χ^2's(1)≤ 2.87, *p's*≥.09; RMSEAs≤.15 (90%CIs=[LLs=.000; ULs≤.358]); CFIs≥.94; SRMRs≤.05). In all 8 models, we found total effects of group, i.e., training, on all outcome variables (95%CIs for expected increases in SMT- vs. AT-group=[LLs≥.158]; 95%CIs for expected decreases in SMT- vs. AT-group=[ULs≤-.120]). The subscale cognitive-strategies-and-problem-solving proved to be the most important mediator since we found indirect effects via this subscale for all outcomes (main model and additional model: 95%CIs for expected increases in SMT- vs. AT-group=[LLs≥.004]; 95%CIs for expected decreases in SMT- vs. AT-group=[ULs≤-.078]) except job dissatisfaction. There were direct effects of group, i.e., training, for relaxation after work (95%CI=[LL=0.180]), SRS-total-score (95%CI=[UL=-0.070]), and job dissatisfaction (95%CI=[UL=-0.160]), but not for PSS, VE, trait anger, and SRS subscale reactivity-to-work-overload. We also found a direct effect of group and thus training on the subscale cognitive-strategies-and-problem-solving T2 (95%CIs=[LLs≥.237]). Regarding the further subscales, we found indirect effects via the subscale identification-and-use-of-social-resources for VE and the SRS subscale reactivity-to-work-overload (95%CIs=[ULs≤-.002]) in the expected direction and via the subscale relaxation-abilities for PSS (95%CI=[LL=-0.213]) contrary to our expectations in a negative direction. There were no indirect effects via the subscale adequate-anger-expression-and-assertiveness. We, moreover, found direct effects of group, i.e., training, for all outcome variables (95%CIs for expected increases in SMT- vs. AT-group=[LLs≥.157]; 95%CIs for expected decreases in SMT- vs. AT-group=[ULs≤-.069]) except for trait anger in the main mediation model with the subscale relaxation-abilities as mediator. We also found direct effects of the training on T2 of the ISBF subscales identification-and-use-of-social-resources, and relaxation-ability (95%CIs=[LLs≥.028]), but not on T2 of adequate-anger-expression-and-assertiveness.

4 Supplementary Material 4: Discussion of mediation results of the ISBF subscales relaxation-abilities and adequate-anger-expression-and-assertiveness

We found an indirect effect via the subscale relaxation-abilities on PSS, but contrary to our expectations in a negative direction. Here, a bias due to a ceiling effect is conceivable as both groups displayed very high PSS values at baseline. Notably, in clinical studies in the context of cancer, i.e., in highly stressed population, there is evidence that perceived relaxation-abilities mediate or at least relate to beneficial SMT effects (Antoni et al., 2006; Jensen et al., 2013; Marsland et al., 2020), although not unequivocally (Gudenkauf et al., 2015). Especially in highly stressed populations it is possible that first remedy through relaxation is necessary in order to train other techniques with a clear head. This would increase importance of perceived relaxation-abilities for beneficial SMT effects in highly stressed participants, but not in occupational participants in general. Interestingly, there were no indirect effects via the subscale adequate-anger-expression-and-assertiveness. Given that adequate-anger-expression-and-assertiveness could also be a result of successfully using other coping strategies such as cognitive-strategies-and-problem-solving, we speculate that this subscale is rather an outcome of successful SMTs than a mediator of change. This consideration is in line with cognitive-behavioral interventions being commonly used to reduce anger (Fava et al., 1991; Beck and Fernandez, 1998; Gerzina and Drummond, 2000; Lee and DiGiuseppe, 2018). However, the study in mothers of children newly diagnosed with cancer found SMT-induced increases in perceived assertiveness to mediate psychological health outcomes after SMT (Marsland et al., 2020).

5 References Supplementary Material

Antoni, M.H., Lechner, S.C., Kazi, A., Wimberly, S.R., Sifre, T., Urcuyo, K.R., et al. (2006). How stress management improves quality of life after treatment for breast cancer. *Journal of consulting and clinical psychology* 74(6)**,** 1143-1152. doi: 10.1037/0022-006X.74.6.1143.

Beck, R., and Fernandez, E. (1998). Cognitive-behavioral therapy in the treatment of anger: A meta-analysis. *Cognitive therapy and research* 22(1)**,** 63-74. doi: 10.1023/A:1018763902991.

Fava, M., Litrman, A., Halperin, P., Pratr, E., Drews, F.R., Oleshansky, M., et al. (1991). Psychological and Behavioral Benefits of a Stress/Type A Behavior Reduction Program for Healthy Middle-Aged Army Officers. *Psychosomatics* 32(3)**,** 337-342. doi: 10.1016/S0033-3182(91)72073-2.

Gerzina, M.A., and Drummond, P.D. (2000). A multimodal cognitive-behavioural approach to anger reduction in an occupational sample. *Journal of Occupational and Organizational Psychology* 73(2)**,** 181-194. doi: 10.1348/096317900166976.

Gudenkauf, L.M., Antoni, M.H., Stagl, J.M., Lechner, S.C., Jutagir, D.R., Bouchard, L.C., et al. (2015). Brief cognitive–behavioral and relaxation training interventions for breast cancer: A randomized controlled trial. *Journal of consulting and clinical psychology* 83(4)**,** 677-688. doi: 10.1037/ccp0000020.

Jensen, S.E., Pereira, D.B., Whitehead, N., Buscher, I., McCalla, J., Andrasik, M., et al. (2013). Cognitive–behavioral stress management and psychological well-being in HIV+ racial/ethnic minority women with human papillomavirus. *Health Psychology* 32(2)**,** 227-230. doi: 10.1037/a0028160.

Lee, A.H., and DiGiuseppe, R. (2018). Anger and aggression treatments: a review of meta-analyses. *Current opinion in psychology* 19**,** 65-74. doi: 10.1016/j.copsyc.2017.04.004.

Marsland, A.L., Walsh, C.P., Cleary, J.L., Vaisleib, A.D., Farrell, C., Woods, W.C., et al. (2020). Efficacy of a Stress Management Intervention for Mothers of Children with Cancer. *Journal of Pediatric Psychology* 45(7)**,** 812-824. doi: 10.1093/jpepsy/jsaa058.

Stein, F. (2007). *Psychoendokrinologische Evaluation eines Stressmanagement Trainings im betrieblichen Umfeld einer Betriebskrankenkasse.* Cuvillier Verlag.

Wirtz, P.H., Thomas, L., Domes, G., Penedo, F.J., Ehlert, U., and Nussbeck, F.W. (2013). Psychoendocrine validation of a short measure for assessment of perceived stress management skills in different non-clinical populations. *Psychoneuroendocrinology* 38(4)**,** 572-586. doi: 10.1016/j.psyneuen.2012.07.017.
